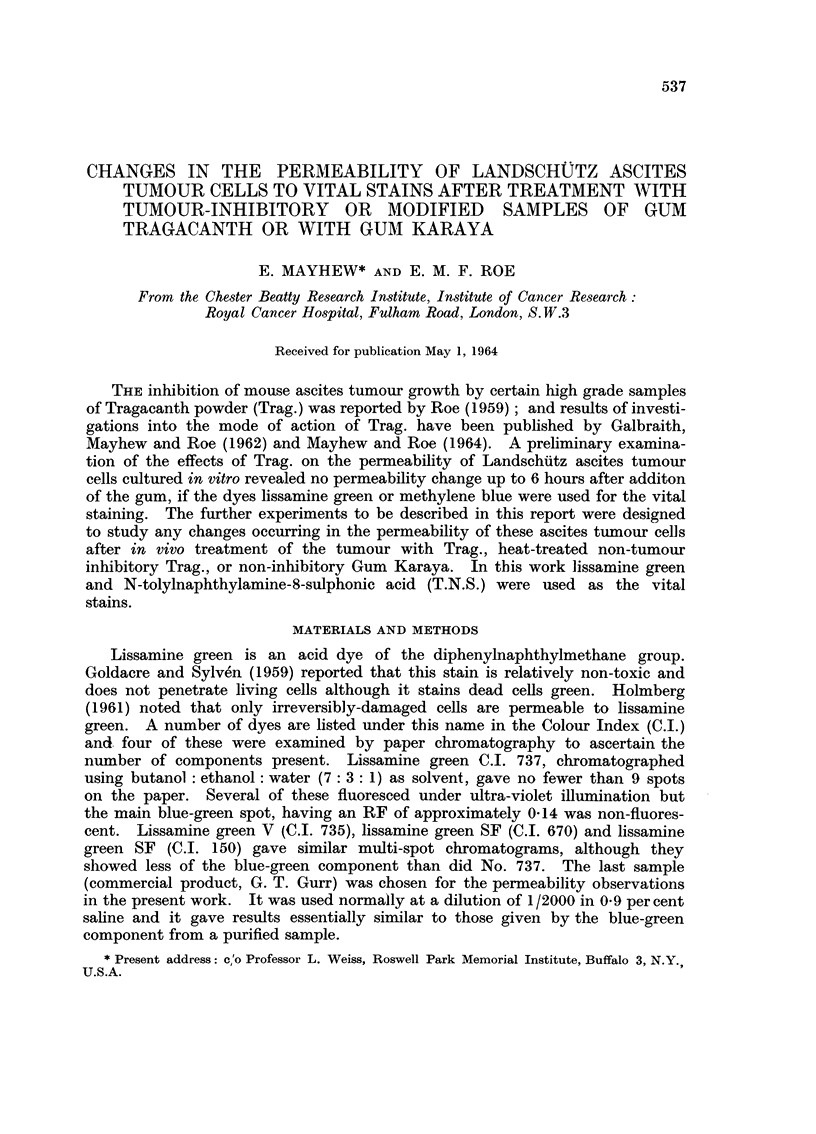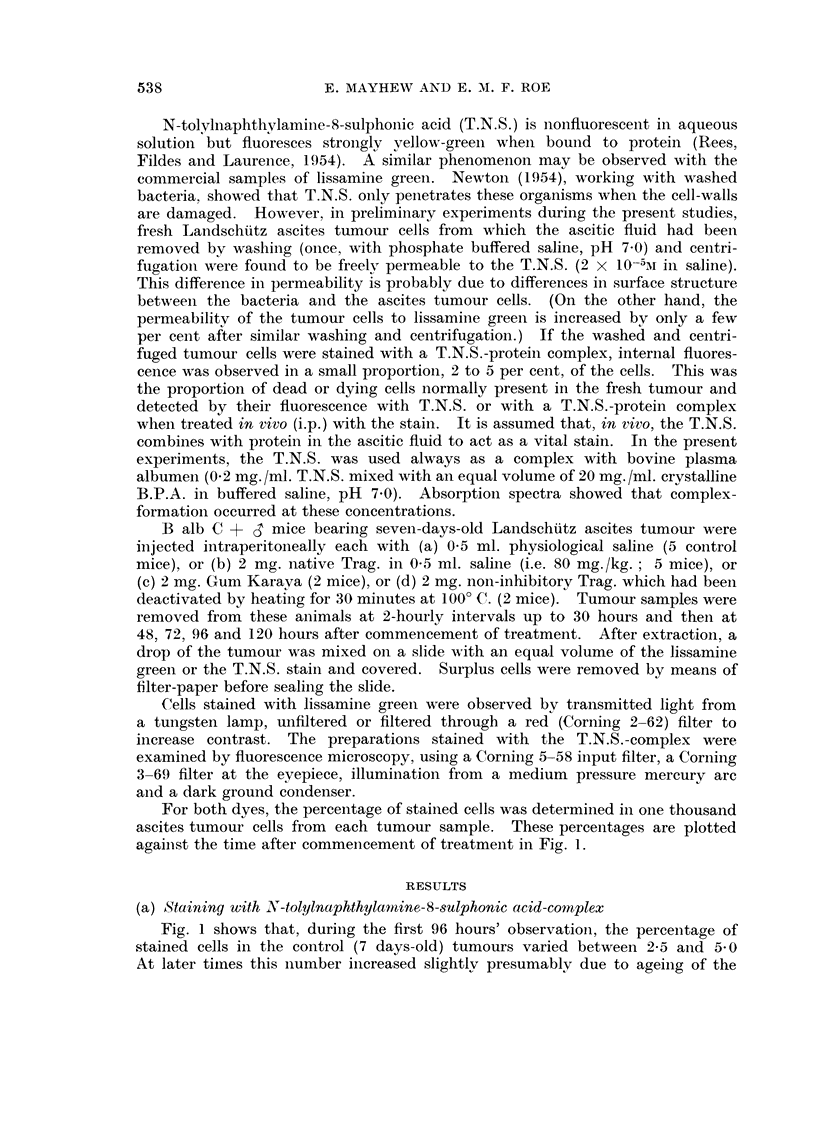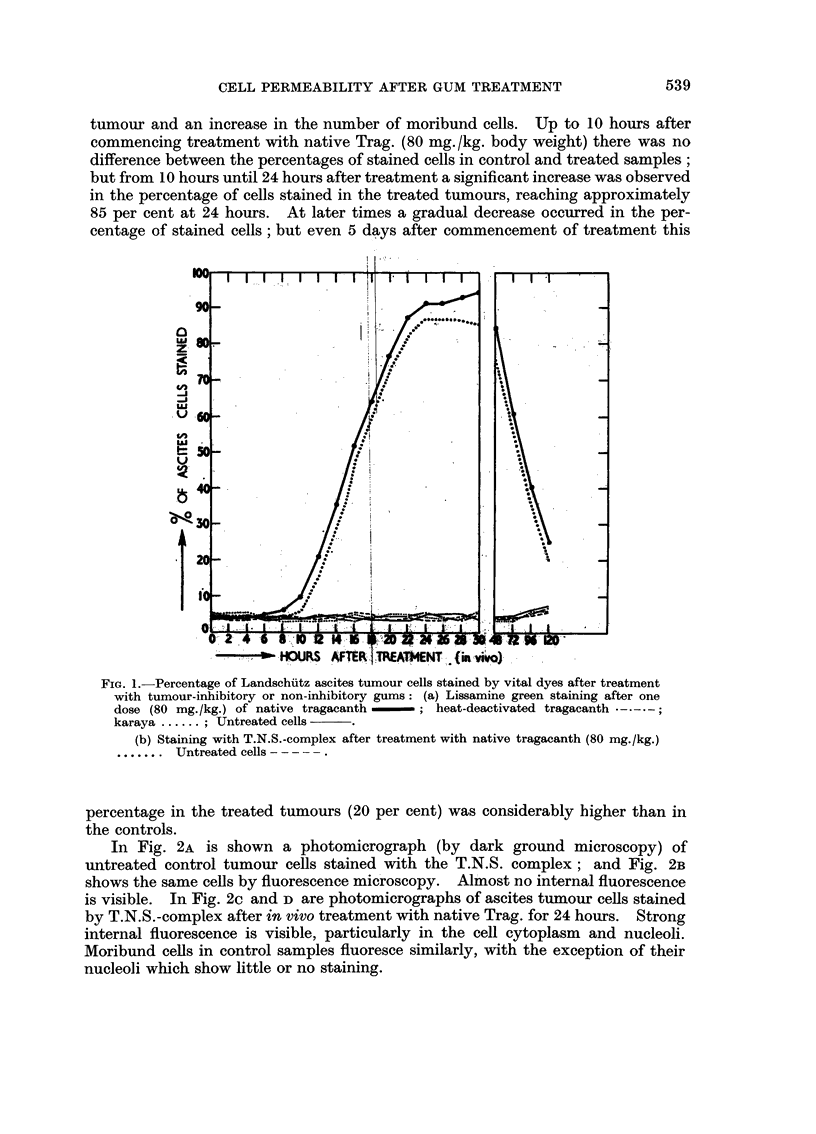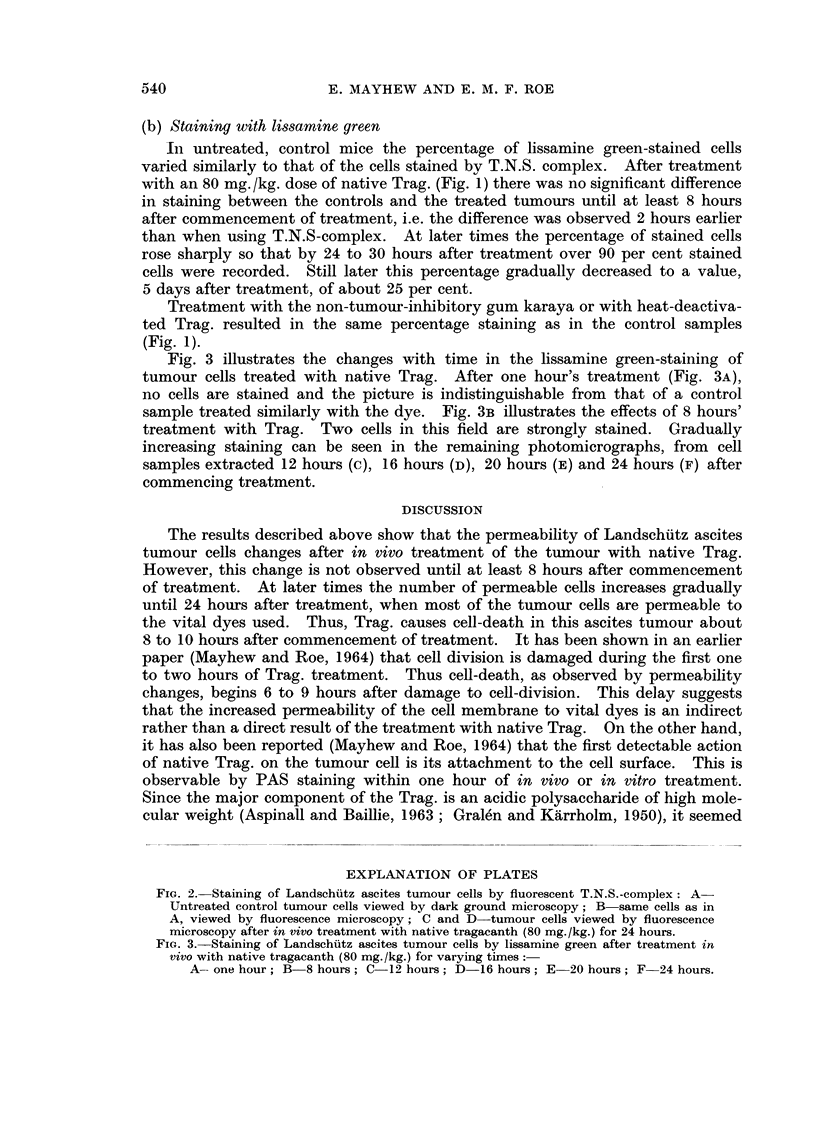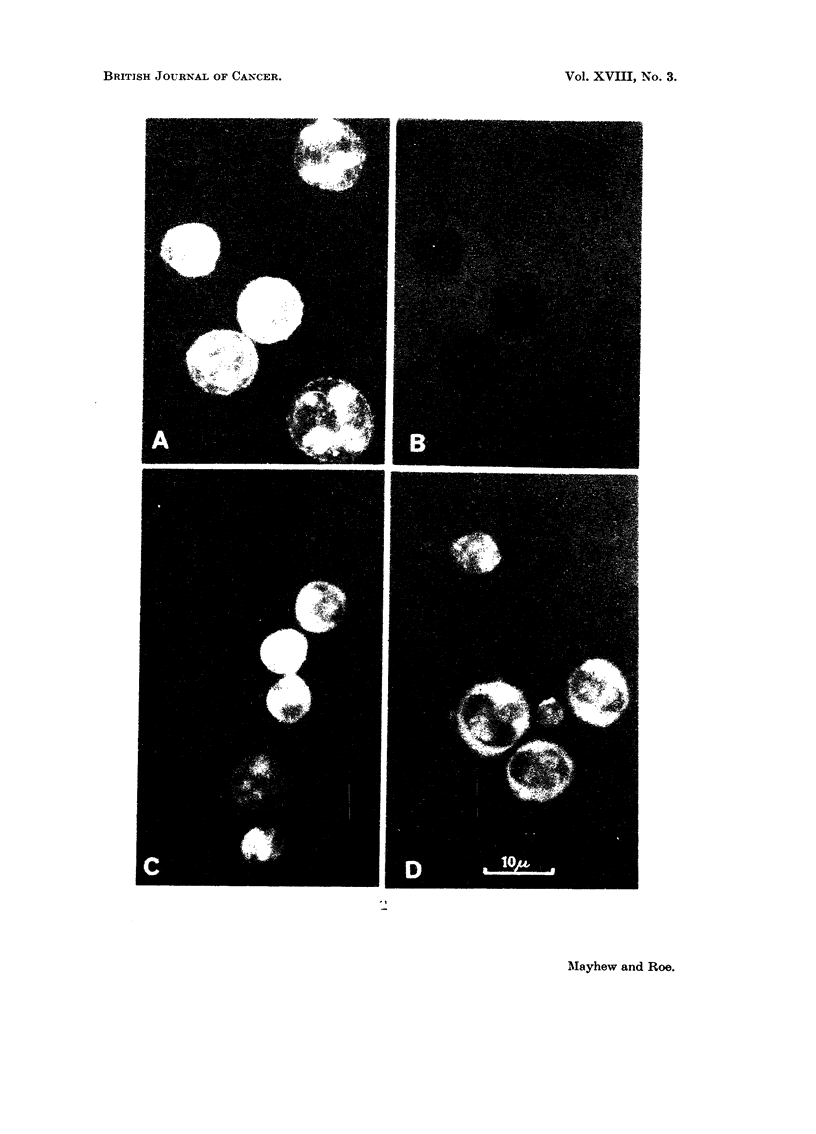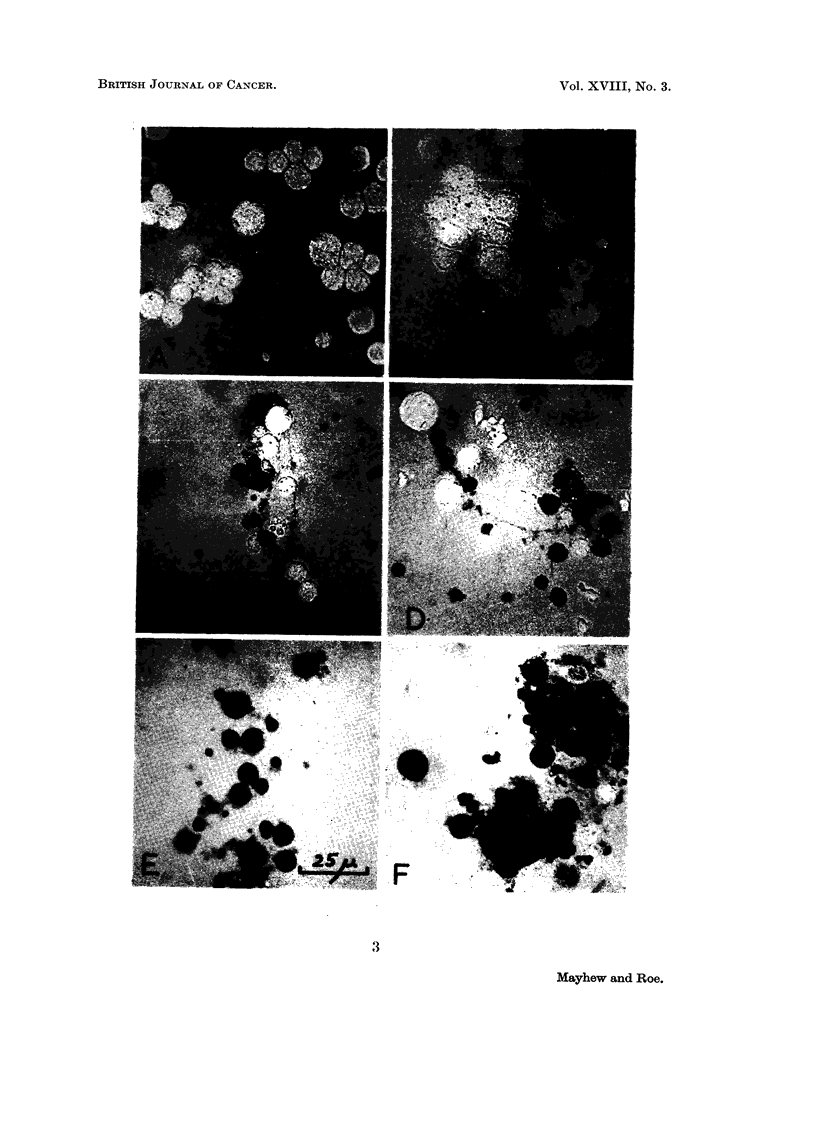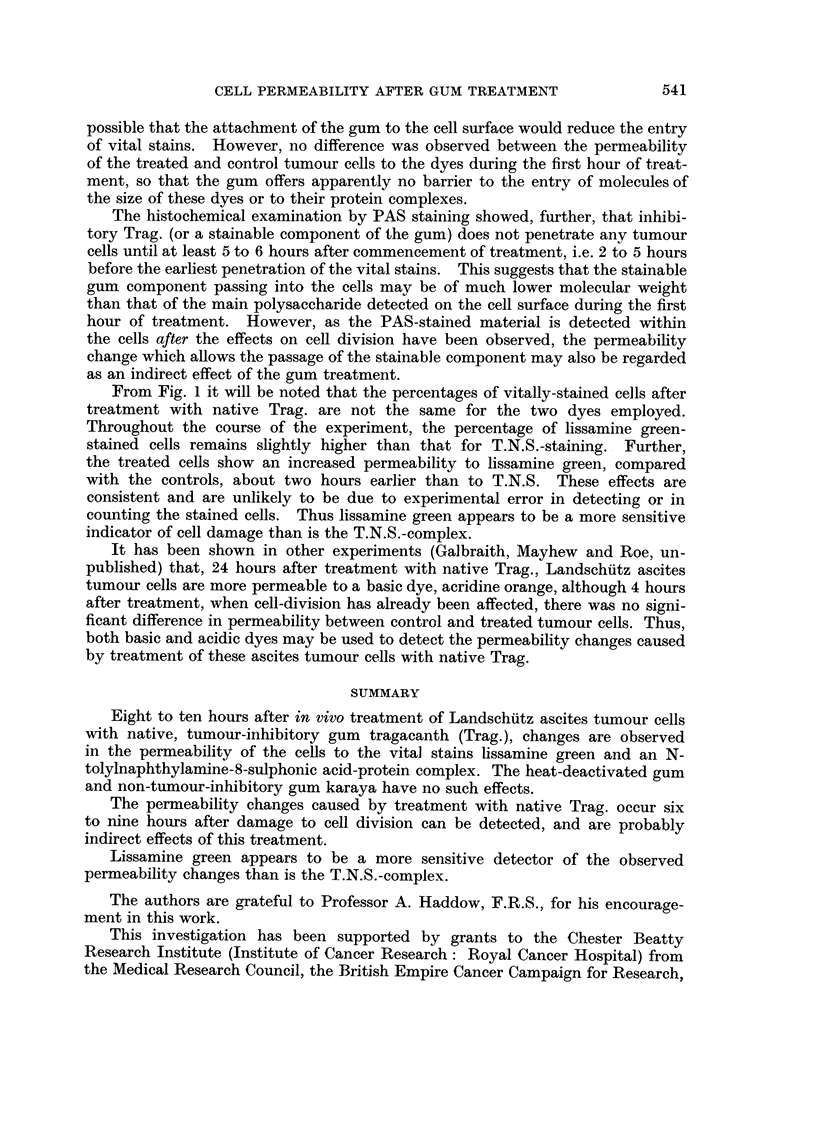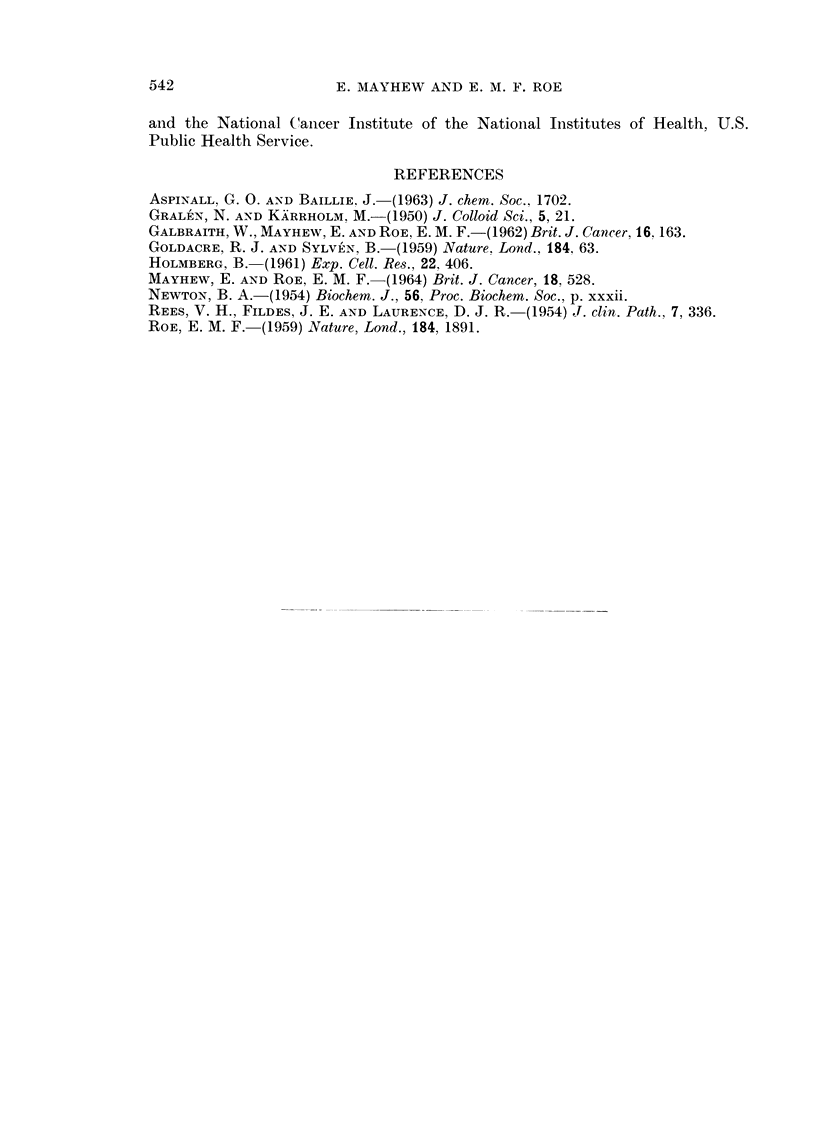# Changes in the Permeability of Landschütz Ascites Tumour Cells to Vital Stains after Treatment with Tumour-Inhibitory or modified Samples of Gum Tragacanth or with Gum Karaya

**DOI:** 10.1038/bjc.1964.61

**Published:** 1964-09

**Authors:** E. Mayhew, E. M. F. Roe

## Abstract

**Images:**


### CHANGES IN THE PERMEABILITY OF LANDSCHÜTZ ASCITES TUMOUR CELLS TO VITAL STAINS AFTER TREATMENT WITH TUMOUR-INHIBITORY OR MODIFIED SAMPLES OF GUM TRAGACANTH OR WITH GUM KARAYA

E. MAYHEW* AND E. M. F. ROE

*From the Chester Beatty Research Institute, Institute of Cancer Research : Royal Cancer Hospital, Fulham Road, London, S.W.3*

Received for publication May 1, 1964

THE inhibition of mouse ascites tumour growth by certain high grade samples of Tragacanth powder (Trag.) was reported by Roe (1959) ; and results of investigations into the mode of action of Trag. have been published by Galbraith, Mayhew and Roe (1962) and Mayhew and Roe (1964). A preliminary examination of the effects of Trag. on the permeability of Landschütz ascites tumour cells cultured *in vitro* revealed no permeability change up to 6 hours after additon of the gum, if the dyes lissamine green or methylene blue were used for the vital staining. The further experiments to be described in this report were designed to study any changes occurring in the permeability of these ascites tumour cells after *in vivo* treatment of the tumour with Trag., heat-treated non-tumour inhibitory Trag., or non-inhibitory Gum Karaya. In this work lissamine green and N-tolylnaphthylamine-8-sulphonic acid (T.N.S.) were used as the vital stains.

### MATERIALS AND METHODS

Lissamine green is an acid dye of the diphenylnaphthylmethane group. Goldacre and Sylvén (1959) reported that this stain is relatively non-toxic and does not penetrate living cells although it stains dead cells green. Holmberg (1961) noted that only irreversibly-damaged cells are permeable to lissamine green. A number of dyes are listed under this name in the Colour Index (C.I.) and four of these were examined by paper chromatography to ascertain the number of components present. Lissamine green C.I. 737, chromatographed using butanol : ethanol : water (7 : 3 : 1) as solvent, gave no fewer than 9 spots on the paper. Several of these fluoresced under ultra-violet illumination but the main blue-green spot, having an RF of approximately 0·14 was non-fluorescent. Lissamine green V (C.I. 735), lissamine green SF (C.I. 670) and lissamine green SF (C.I. 150) gave similar multi-spot chromatograms, although they showed less of the blue-green component than did No. 737. The last sample (commercial product, G. T. Gurr) was chosen for the permeability observations in the present work. It was used normally at a dilution of 1/2000 in 0·9 per cent saline and it gave results essentially similar to those given by the blue-green component from a purified sample.

* Present address: c/o Professor L. Weiss, Roswell Park Memorial Institute, Buffalo 3, N.Y., U.S.A.

N-tolylnaphthylamine-8-sulphonic acid (T.N.S.) is nonfluorescent in aqueous solution but fluoresces strongly yellow-green when bound to protein (Rees, Fildes and Laurence, 1954). A similar phenomenon may be observed with the commercial samples of lissamine green. Newton (1954), working with washed bacteria, showed that T.N.S. only penetrates these organisms when the cell-walls are damaged. However, in preliminary experiments during the present studies, fresh Landschütz ascites tumour cells from which the ascitic fluid had been removed by washing (once, with phosphate buffered saline, pH 7·0) and centrifugation were found to be freely permeable to the T.N.S. ($2 \times 10^{-5}$M in saline). This difference in permeability is probably due to differences in surface structure between the bacteria and the ascites tumour cells. (On the other hand, the permeability of the tumour cells to lissamine green is increased by only a few per cent after similar washing and centrifugation.) If the washed and centrifuged tumour cells were stained with a T.N.S.-protein complex, internal fluorescence was observed in a small proportion, 2 to 5 per cent, of the cells. This was the proportion of dead or dying cells normally present in the fresh tumour and detected by their fluorescence with T.N.S. or with a T.N.S.-protein complex when treated *in vivo* (i.p.) with the stain. It is assumed that, *in vivo*, the T.N.S. combines with protein in the ascitic fluid to act as a vital stain. In the present experiments, the T.N.S. was used always as a complex with bovine plasma albumen (0·2 mg./ml. T.N.S. mixed with an equal volume of 20 mg./ml. crystalline B.P.A. in buffered saline, pH 7·0). Absorption spectra showed that complex-formation occurred at these concentrations.

B alb C + ♂ mice bearing seven-days-old Landschütz ascites tumour were injected intraperitoneally each with (a) 0·5 ml. physiological saline (5 control mice), or (b) 2 mg. native Trag. in 0·5 ml. saline (i.e. 80 mg./kg. ; 5 mice), or (c) 2 mg. Gum Karaya (2 mice), or (d) 2 mg. non-inhibitory Trag. which had been deactivated by heating for 30 minutes at 100° C. (2 mice). Tumour samples were removed from these animals at 2-hourly intervals up to 30 hours and then at 48, 72, 96 and 120 hours after commencement of treatment. After extraction, a drop of the tumour was mixed on a slide with an equal volume of the lissamine green or the T.N.S. stain and covered. Surplus cells were removed by means of filter-paper before sealing the slide.

Cells stained with lissamine green were observed by transmitted light from a tungsten lamp, unfiltered or filtered through a red (Corning 2–62) filter to increase contrast. The preparations stained with the T.N.S.-complex were examined by fluorescence microscopy, using a Corning 5–58 input filter, a Corning 3–69 filter at the eyepiece, illumination from a medium pressure mercury arc and a dark ground condenser.

For both dyes, the percentage of stained cells was determined in one thousand ascites tumour cells from each tumour sample. These percentages are plotted against the time after commencement of treatment in Fig. 1.

### RESULTS

### (a) *Staining with N-tolylnaphthylamine-8-sulphonic acid-complex*

Fig. 1 shows that, during the first 96 hours' observation, the percentage of stained cells in the control (7 days-old) tumours varied between 2·5 and 5·0 At later times this number increased slightly presumably due to ageing of the tumour and an increase in the number of moribund cells. Up to 10 hours after commencing treatment with native Trag. (80 mg./kg. body weight) there was no difference between the percentages of stained cells in control and treated samples ; but from 10 hours until 24 hours after treatment a significant increase was observed in the percentage of cells stained in the treated tumours, reaching approximately 85 per cent at 24 hours. At later times a gradual decrease occurred in the percentage of stained cells ; but even 5 days after commencement of treatment this

FIG. 1.—Percentage of Landschütz ascites tumour cells stained by vital dyes after treatment with tumour-inhibitory or non-inhibitory gums : (a) Lissamine green staining after one dose (80 mg./kg.) of native tragacanth ▬▬▬ ; heat-deactivated tragacanth -·-·-·- ; karaya ...... ; Untreated cells ———.

(b) Staining with T.N.S.-complex after treatment with native tragacanth (80 mg./kg.) ....... Untreated cells – – – – – – .

percentage in the treated tumours (20 per cent) was considerably higher than in the controls.

In Fig. 2A is shown a photomicrograph (by dark ground microscopy) of untreated control tumour cells stained with the T.N.S. complex ; and Fig. 2B shows the same cells by fluorescence microscopy. Almost no internal fluorescence is visible. In Fig. 2C and D are photomicrographs of ascites tumour cells stained by T.N.S.-complex after *in vivo* treatment with native Trag. for 24 hours. Strong internal fluorescence is visible, particularly in the cell cytoplasm and nucleoli. Moribund cells in control samples fluoresce similarly, with the exception of their nucleoli which show little or no staining.

(b) *Staining with lissamine green*

In untreated, control mice the percentage of lissamine green-stained cells varied similarly to that of the cells stained by T.N.S. complex. After treatment with an 80 mg./kg. dose of native Trag. (Fig. 1) there was no significant difference in staining between the controls and the treated tumours until at least 8 hours after commencement of treatment, i.e. the difference was observed 2 hours earlier than when using T.N.S-complex. At later times the percentage of stained cells rose sharply so that by 24 to 30 hours after treatment over 90 per cent stained cells were recorded. Still later this percentage gradually decreased to a value, 5 days after treatment, of about 25 per cent.

Treatment with the non-tumour-inhibitory gum karaya or with heat-deactivated Trag. resulted in the same percentage staining as in the control samples (Fig. 1).

Fig. 3 illustrates the changes with time in the lissamine green-staining of tumour cells treated with native Trag. After one hour's treatment (Fig. 3A), no cells are stained and the picture is indistinguishable from that of a control sample treated similarly with the dye. Fig. 3B illustrates the effects of 8 hours' treatment with Trag. Two cells in this field are strongly stained. Gradually increasing staining can be seen in the remaining photomicrographs, from cell samples extracted 12 hours (C), 16 hours (D), 20 hours (E) and 24 hours (F) after commencing treatment.

### DISCUSSION

The results described above show that the permeability of Landschütz ascites tumour cells changes after *in vivo* treatment of the tumour with native Trag. However, this change is not observed until at least 8 hours after commencement of treatment. At later times the number of permeable cells increases gradually until 24 hours after treatment, when most of the tumour cells are permeable to the vital dyes used. Thus, Trag. causes cell-death in this ascites tumour about 8 to 10 hours after commencement of treatment. It has been shown in an earlier paper (Mayhew and Roe, 1964) that cell division is damaged during the first one to two hours of Trag. treatment. Thus cell-death, as observed by permeability changes, begins 6 to 9 hours after damage to cell-division. This delay suggests that the increased permeability of the cell membrane to vital dyes is an indirect rather than a direct result of the treatment with native Trag. On the other hand, it has also been reported (Mayhew and Roe, 1964) that the first detectable action of native Trag. on the tumour cell is its attachment to the cell surface. This is observable by PAS staining within one hour of *in vivo* or *in vitro* treatment. Since the major component of the Trag. is an acidic polysaccharide of high molecular weight (Aspinall and Baillie, 1963 ; Gralén and Kärrholm, 1950), it seemed

---

### EXPLANATION OF PLATES

FIG. 2.—Staining of Landschütz ascites tumour cells by fluorescent T.N.S.-complex : A—Untreated control tumour cells viewed by dark ground microscopy ; B—same cells as in A, viewed by fluorescence microscopy ; C and D—tumour cells viewed by fluorescence microscopy after *in vivo* treatment with native tragacanth (80 mg./kg.) for 24 hours.

FIG. 3.—Staining of Landschütz ascites tumour cells by lissamine green after treatment *in vivo* with native tragacanth (80 mg./kg.) for varying times :—

A— one hour ; B—8 hours ; C—12 hours ; D—16 hours ; E—20 hours ; F—24 hours.

2

Mayhew and Roe.

3

Mayhew and Roe.

possible that the attachment of the gum to the cell surface would reduce the entry of vital stains. However, no difference was observed between the permeability of the treated and control tumour cells to the dyes during the first hour of treatment, so that the gum offers apparently no barrier to the entry of molecules of the size of these dyes or to their protein complexes.

The histochemical examination by PAS staining showed, further, that inhibitory Trag. (or a stainable component of the gum) does not penetrate any tumour cells until at least 5 to 6 hours after commencement of treatment, i.e. 2 to 5 hours before the earliest penetration of the vital stains. This suggests that the stainable gum component passing into the cells may be of much lower molecular weight than that of the main polysaccharide detected on the cell surface during the first hour of treatment. However, as the PAS-stained material is detected within the cells *after* the effects on cell division have been observed, the permeability change which allows the passage of the stainable component may also be regarded as an indirect effect of the gum treatment.

From Fig. 1 it will be noted that the percentages of vitally-stained cells after treatment with native Trag. are not the same for the two dyes employed. Throughout the course of the experiment, the percentage of lissamine green-stained cells remains slightly higher than that for T.N.S.-staining. Further, the treated cells show an increased permeability to lissamine green, compared with the controls, about two hours earlier than to T.N.S. These effects are consistent and are unlikely to be due to experimental error in detecting or in counting the stained cells. Thus lissamine green appears to be a more sensitive indicator of cell damage than is the T.N.S.-complex.

It has been shown in other experiments (Galbraith, Mayhew and Roe, unpublished) that, 24 hours after treatment with native Trag., Landschütz ascites tumour cells are more permeable to a basic dye, acridine orange, although 4 hours after treatment, when cell-division has already been affected, there was no significant difference in permeability between control and treated tumour cells. Thus, both basic and acidic dyes may be used to detect the permeability changes caused by treatment of these ascites tumour cells with native Trag.

### SUMMARY

Eight to ten hours after *in vivo* treatment of Landschütz ascites tumour cells with native, tumour-inhibitory gum tragacanth (Trag.), changes are observed in the permeability of the cells to the vital stains lissamine green and an N-tolylnaphthylamine-8-sulphonic acid-protein complex. The heat-deactivated gum and non-tumour-inhibitory gum karaya have no such effects.

The permeability changes caused by treatment with native Trag. occur six to nine hours after damage to cell division can be detected, and are probably indirect effects of this treatment.

Lissamine green appears to be a more sensitive detector of the observed permeability changes than is the T.N.S.-complex.

The authors are grateful to Professor A. Haddow, F.R.S., for his encouragement in this work.

This investigation has been supported by grants to the Chester Beatty Research Institute (Institute of Cancer Research : Royal Cancer Hospital) from the Medical Research Council, the British Empire Cancer Campaign for Research, and the National Cancer Institute of the National Institutes of Health, U.S. Public Health Service.

### REFERENCES

ASPINALL, G. O. AND BAILLIE, J.—(1963) *J. chem. Soc.*, 1702.
GRALÉN, N. AND KÄRRHOLM, M.—(1950) *J. Colloid Sci.*, **5**, 21.
GALBRAITH, W., MAYHEW, E. AND ROE, E. M. F.—(1962) *Brit. J. Cancer*, **16**, 163.
GOLDACRE, R. J. AND SYLVÉN, B.—(1959) *Nature, Lond.*, **184**, 63.
HOLMBERG, B.—(1961) *Exp. Cell. Res.*, **22**, 406.
MAYHEW, E. AND ROE, E. M. F.—(1964) *Brit. J. Cancer*, **18**, 528.
NEWTON, B. A.—(1954) *Biochem. J.*, **56**, *Proc. Biochem. Soc.*, p. xxxii.
REES, V. H., FILDES, J. E. AND LAURENCE, D. J. R.—(1954) *J. clin. Path.*, **7**, 336.
ROE, E. M. F.—(1959) *Nature, Lond.*, **184**, 1891.